# Mental health and caregiving experiences of family carers supporting people with psychosis

**DOI:** 10.1017/S2045796020001067

**Published:** 2021-01-08

**Authors:** J. Sin, J. Elkes, R. Batchelor, C. Henderson, S. Gillard, L.A. Woodham, T. Chen, A. Aden, V. Cornelius

**Affiliations:** 1 School of Health Sciences, City, University of London, Myddelton Street Building, 1 Myddelton Street, London EC1R 1UW, England, UK; 2 Imperial Clinical Trials Unit, School of Public Health, Imperial College London, Stadium House, 68 Wood Lane, London W12 7RH, England, UK; 3 Population Health Research Institute, St George's, University of London, Cranmer Terrace, London SW17 0RE, England, UK; 4 Health Service & Population Research Department, King's College London, Institute of Psychiatry, Psychology & Neuroscience, De Crespigny Park, London SE5 8AF, England, UK; 5 Centre for Technology in Education, St George's, University of London, Cranmer Terrace, London SW17 0RE, England, UK; 6 Department of Clinical Sciences, Liverpool School of Tropical Medicine, Pembroke Place, Liverpool L3 5QA, England, UK

**Keywords:** Caregiving, family carers, psychosis, public mental health, quality of life, wellbeing

## Abstract

**Aims:**

Family carers supporting an individual with psychosis often experience poorer mental health, however, little is known about specific risk factors among these carers. We investigated the associations between demographic, caregiving characteristics and mental health outcomes in family carers supporting an individual with psychosis and compared carers' outcomes with general population norms.

**Methods:**

We analysed baseline data from the COPe-support randomised controlled trial of online psychoeducation and peer support for adult carers supporting an individual with psychosis between 2018 and 2020. We collected carers' demographic and health outcome data, including wellbeing using Warwick-Edinburgh Mental Wellbeing Scale (WEMWBS as primary outcome), quality of life using EQ-5D-5L and caregiving experience assessed with Experience of Caregiving Inventory. We tested associations between carers' demographic and caregiving characteristics for each outcome in turn and meta-analysed carers' WEMWBS and EQ-5D-5L with Health Survey England (HSE) general population data from 2016 and 2017, respectively.

**Results:**

The 407 carers of people with psychosis had a mean WEMWBS score of 42.2 (s.d. 9.21) and their overall weighted pooled WEMWBS score was 7.3 (95% confidence interval (CI) −8.6 to −6.0, *p* < 0.01) lower than the HSE general population sample, indicating carers have poorer mental wellbeing by more than double the minimum clinically important difference of 3 points on WEMWBS. Among all caring relationships, partners had poorer wellbeing compared to parents with lower WEMWBS score (−6.8, −16.9 to 3.3, *p* = 0.03). Single carers had significantly poorer wellbeing (−3.6, −5.6 to −1.5, *p* < 0.01) and a more negative caregiving experience than those who were cohabiting. Spending more than 35 h per week caregiving increased carers' negative experience significantly (*p* = 0.01).

**Conclusion:**

Carers of people with psychosis have poorer mental health than non-carers. Partners, lone carers and those spending more than 35 h per week on caring were found to be most at risk of poor mental health. Based on the results, we advocate that the details of carers for individuals with psychosis should be added to the existing carers or severe mental illness registers at all general practitioner surgeries and for their wellbeing screened routinely. Future large-scale prospective studies are needed to develop a predictive model to determine risk factors, hence to aid early identification of carers' support needs. Such understandings are also useful to inform tailored intervention development.

## Introduction

It is estimated that approximately 1.5 million people in the UK are caring for a family member or friend with a mental illness (The Schizophrenia Commission, 2012; NICE, 2014; Carers Trust, 2019). Psychotic disorders or psychosis are recognised as among the most common severe mental illness (SMI), often with worst outcomes (The Schizophrenia Commission, 2012; NICE, 2014). ‘Psychosis’ could be regarded as a broad category of major mental health conditions (such as schizophrenia, schizoaffective disorder and delusional disorder) that have psychotic symptoms as its hallmarks. Psychotic symptoms can cause significant distress in an individual and far-reaching impacts on their perception, thoughts, mood, behaviour and functioning. Often, people with psychosis require long-term treatment and support across a range of life domains, including emotional support, and financial and practical assistance for daily living activities (The Schizophrenia Commission, 2012; Sin and Norman, 2013; NICE, 2014; Sin *et al*., 2017). The importance of relatives and friends providing a supportive role – commonly referred to as family carers (or carers as referred to thereafter) – is well established. Individuals who receive support and care from their familial networks have a better prognosis and enhanced quality of life (QoL) (Pharoah *et al*., 2010; Sin *et al*., 2016).

The load and responsibility of caregiving can cause high levels of distress affecting the mental health of the carers themselves (Singleton *et al*., 2002; Smith *et al*., 2014; Stansfeld *et al*., 2014). Over the last decade, Health Survey England (HSE) (Bridges, 2012) and Adult Psychiatric Morbidity Surveys (APMS) (Smith *et al*., 2014; Stansfeld *et al*., 2014) have published some reports focusing on carers' mental health, among their regular English adult population survey reports (e.g. McManus *et al*., 2016; NHS Digital HSE, 2016, 2017). These repeatedly reported that carers of individuals with ill health, disability or frailty (i.e. general carers) have poorer wellbeing than non-carers in the general population. A HSE 2012 report investigating carers' mental wellbeing identified that general carers who provided caregiving for 10 or more hours per week (hpw) (*n* = 227) had the lowest mental wellbeing scores, compared to those providing up to 9 h (*n* = 560) or no care (*n* = 137), respectively (Bridges, 2012). Furthermore, carers' mental health morbidities are known to be correlated with the amount of care they provide. In an analysis of APMS 2007 general population survey data, 25% (*n* = 1883) of participants, out of the total 7304 people, identified themselves as regular general carers supporting a family member or friend (Smith *et al*., 2014). The carers were found to have poorer mental health than non-carers and those caregiving more than 20 hpw had a twofold increase in mental distress symptomatology (Smith *et al*., 2014).

Comparing with caring for a loved one with a physical illness, supporting an individual with psychosis where comorbid physical health problems are common, is known to be much more demanding and stressful (Singleton *et al*., 2002; The Schizophrenia Commission, 2012; NICE, 2014; Steptoe *et al*., 2015). Research evidence further suggests that poor mental health in carers can negatively impact their caregiving capacity (Bebbington and Kuipers, 1994; Szmukler *et al*., 1996; Steptoe *et al*., 2015), rendering them less likely to engage in caring for their loved ones or more likely to exhibit critical or hostile behaviour towards the cared-for persons (CfP) (Szmukler *et al*., 1996; Cooper *et al*., 2010). In turn, high expressed emotion (EE), i.e. critical attitude or over-involvement within the family environment, has long been shown to increase risk of relapses in patients by three to fourfold (Bebbington and Kuipers, 1994), potentially leading to a vicious cycle of poor health and QoL for all concerned. Hence in the last decade, the UK government has published multiple policies and strategies aimed at identifying carers to provide them with support and intervention as early as possible (DOH, 2008, 2014; NICE, 2014; Yesufu-Udechuku *et al*., 2015). Although healthcare practitioners in primary and mental health services are best placed to identify and provide support for the carer population, in particular for those supporting an individual with psychosis, research evidence identifies an ongoing implementation gap (Sin *et al*., 2018; HQIP & Royal College of Psychiatrists, 2016).

Most prior studies on carers’ mental wellbeing and other health outcomes have focused on those who support a loved one with a long-term illness or dementia, but not specific to psychosis. Systematic reviews on carers of people with psychosis report approximately 80% of the study participants were females, especially mothers for their adult child suffering from psychosis (Sin and Norman, 2013; Yesufu-Udechuku *et al*., 2015; Sin *et al*., 2017). Nearly half of these carers were provided over 32 hpw caregiving (Roick *et al*., 2007), potentially increasing their health morbidities. To date, little is known about other carers with a different relationship to the CfP, such as partners, children or siblings. Similarly, beyond time spent on caregiving, few studies have investigated other factors affecting carers' mental health outcomes. The paucity of high-quality research translates into a lack of routine identification of carers and monitoring of their health outcomes. In order to establish how carers of people with psychosis fare in terms of mental wellbeing and QoL, we compared their outcomes with those of their counterparts in the general population. To further understand plausible risk factors associated with carers' mental health outcomes for timely identification and provision of tailored interventions, we investigated a range of demographic and caregiving factors among carers.

## Methods

### Study population and design

We conducted analyses of baseline data from an online randomised controlled trial (RCT) targeting psychosis carers. The RCT was designed to evaluate a multi-component eHealth intervention, called COPe-support (Sin *et al*., 2019), for carers of individuals with psychosis and took place between 2018 and 2020, as described elsewhere (Sin *et al*., 2020). We recruited carers who have a significant emotional bond with the CfP (as defined by the NICE guideline) (NICE, 2014) and have at least weekly contact in any format including face-to-face meetings and remote interactions. The RCT recruitment activities took place across 30 NHS mental healthcare trusts and various voluntary carer services in England. Both the carers and their CfP were required to be residing in England during the study period (Sin *et al*., 2020). All eligible carers were accepted into the RCT regardless of any support or services they received. The baseline data collected from the RCT participants were utilised in this study.

We extracted data from HSE samples on general population mental wellbeing (NHS Digital HSE, 2016) and QoL data (NHS Digital HSE, 2017), to compare to the carers in the study. HSE is a series of annual, household surveys that gather cross-sectional data at the household and individual level. It uses a multi-stage stratified random probability sampling method to obtain a sample representative of the general population living in private households in England, based on postcode sector (see Craig *et al*., 2014 for further details of HSE sampling and data collection procedures). Each annual survey includes core questions and measurements concerning health conditions which are the same each year, in addition to year-specific questions that focus on particular health outcomes (Craig *et al*., 2014). These include the measurement of mental wellbeing in HSE 2016 and QoL in HSE 2017 (NHS Digital HSE, 2016, 2017).

### Data collection

We collected baseline data from the participants through our online platform. These included carers' demographic and 12 caregiving-related characteristics: age of carers and CfP; gender of the carers and their CfP; carer's ethnicity as White or Black, Asian and minority ethnic (BAME); carers' relationship with the CfP (parent, partner, child or sibling, or other relatives/close friend); carers' employment status; highest education level achieved; marital status; the specific type of psychotic disorder the CfP suffered; time since illness onset in the CfP; living with the CfP or not; and hours spent caregiving per week.

### Outcomes for population comparison

The primary health outcome was the carers' mental wellbeing, measured with the Warwick-Edinburgh Mental Wellbeing Scale (WEMWBS) (Tennant *et al*., 2007). WEMWBS scores range from 14 (minimum) to 70 (maximum); the higher the score the better the individual's mental wellbeing and a change of 3 points in WEMWBS represents the minimum clinically important difference (MCID) (Maheswaran *et al*., 2012). We also examined carers' QoL measured by EQ-5D-5L (Szende *et al*., 2014). EQ-5D-5L includes two parts: the visual analogue scale (VAS) which ranges from 0 (the worst health) to 100 (the best health) reflecting the individual's own judgement and the index value of overall health-related QoL (Szende *et al*., 2014). The single index value ranges from 0 (dead) to 1 (full health) reflecting how good or bad the individual's health state is according to the preferences of the general population of that country (van Hout *et al*., 2012). Both WEMWBS and EQ-5D-5L have been widely used in epidemiological studies, including the Health Surveys in England in 2016 and 2017 (NHS Digital HSE, 2016, 2017) from which we drew our comparison data as published population-level data.

### Health outcomes in the carer sample only

We investigated other caregiving-related health outcomes which are known to be associated with carers' mental wellbeing, including carers' knowledge of mental health, appraisal of caregiving experience, carer-specific wellbeing and perceived support and EE (Bebbington and Kuipers, 1994; Szmukler *et al*., 1996; Singleton *et al*., 2002; Bridges, 2012; Smith *et al*., 2014; Sin *et al*., 2017). We assessed carers' mental health knowledge with the Mental Health Knowledge Schedule (MAKS) (Evans-Lacko *et al*., 2010). MAKS scores range from 6 to 30; higher score indicates better knowledge of mental health (Evans-Lacko *et al*., 2010; Henderson and Thornicroft, 2013). We measured carers' wellbeing and perceived support using Carer Wellbeing and Support scale (CWS), which was specifically designed for the carer populations (Quirk *et al*., 2012). Higher wellbeing scale total (range: 0–128) indicates better carer wellbeing; while higher support scores (reverse scoring used and total range: 0–51) indicates lower satisfaction with support received. Appraisal of caregiving was assessed using the Experience of Caregiving Inventory (ECI) (Szmukler *et al*., 1996) which produces two subscale scores: a negative subtotal ranging from 0 to 208 (higher scores indicate poorer negative appraisal of the caring situation, covering problems with services, stigma and dependency); and a positive subtotal from 0 to 56 (higher scores indicate better positive experience) (Szmukler *et al*., 1996; Joyce *et al*., 2000). Lastly, we assessed carers' EE with Family Questionnaire (FQ), with higher scores indicating worse EE (range: 10–80) (Wiedemann *et al*., 2002).

### Ethics statement

Participants provided informed consent on our online platform for participation in the RCT of COPe-support. Participants received a goodwill payment (an online voucher) for their participation and provision of baseline data. The RCT has been reviewed and approved by South Central – Oxford C Research Ethics Committee (Reference: 18/SC/0104) and Health Research Authority (Reference: IRAS 240005).

### Statistical analysis

The analysis began with computing the demographic and outcome measures (as aforementioned) using appropriate measures to describe central tendency and spread for continuous variables, and contingency tables for categorical variables. We examined associations and relationships between health outcomes through graphical displays, using Pearson's correlation statistic, 95% confidence interval (CI) and *p* value to assess the direction and strength of the correlation. We explored the relationships between demographic characteristics for each outcome, in turn, using linear regression. The intention of the analysis was to explore the association between demographic and caregiving variables so all 12 covariates were included regardless of significance. The functional forms of covariates in the regression models were determined using external literature and visual plots. Interactions between carer characteristics and outcomes were pre-specified using data from published literature (on carers in general) and were: gender and age; relationship type and age of CfP; gender and time spent on care; and the relationship between carer and CfP with time spent on care (Bebbington and Kuipers, 1994; Szmukler *et al*., 1996; Singleton *et al*., 2002; Cooper *et al*., 2010; Bridges, 2012; Smith *et al*., 2014; Sin *et al*., 2017). We included these interactions if there was evidence of their importance *p* < 0.20. We evaluated the model assumptions using residual analysis. We used STATA version 15 (StataCorp., 2017).

To evaluate the difference in WEMWBS scores between our carer sample and the HSE 2016 general population data (NHS Digital HSE, 2016) we conducted a random-effects meta-analysis pooling the mean and standard deviations from each age category across the two populations. For this comparison, HSE wellbeing data were restricted to age ranges that matched those of our carer sample of this study. Standard deviations were estimated using the weighted sample and standard errors available in published reports. Weighted mean difference (WMD) was chosen instead of standardised mean difference (SMD) for interpretability. We used the same method to compare data on EQ-5D index value score for the carer sample with general population data from HSE 2017 survey data, grouped into corresponding age ranges (NHS Digital HSE, 2017).

## Results

### Carer sample characteristics

The RCT comprised 407 carers, out of 464 who gave informed consent and also provided baseline data. Table 1 summarises the demographic data of the carers. The mean age of carers was 53 years and the mean age of the CfP was 35 years. The majority of the carers were White, while 48 (11.8%) self-identifying as BAME, of which one-third (4.2%) described themselves as Black. A majority of participants were female and of these, a further majority cared for a male person. Parents comprised the majority of the participants, followed by partners, while children/siblings or other relatives/close friends formed the remainder (15.2%). Just over half of the participants were in work or education; while nearly a quarter were retired and one-fifth were not currently in work. Most carers described themselves as married or cohabiting, while one-third were single. About half of the participants lived with the CfP. About 40% of carers spent >35 hpw on caregiving; equivalent to a full-time vocational commitment.

**Table 1.** Summary of demographics and caregiving-related characteristics of carers

Carer characteristics (*N* = 407)		
Age, Mean (s.d.)	53.1	(13.2)
Gender	*n*	%
Male	77	18.9
Female	330	81.1
Ethnicity		
White	359	88.2
BAME	48	11.8
Employment status		
Work/Education	224	55.1
Unemployed/Not working	88	21.6
Retired	95	23.3
Highest education level achieved		
Pre-university	127	31.2
Undergraduate/Profession qualification	153	37.6
Postgraduate	87	21.4
Apprenticeship	40	9.8
Marital status		
Married/cohabiting	278	68.3
Single/Divorced/Other	129	31.7
Relationship with CfP		
Parent	258	63.4
Spouse/Partner	87	21.4
Child/Sibling	44	10.8
Friend/Other relatives	18	4.4
Living arrangement		
With CfP	227	55.8
Not with CfP	180	44.2
Duration of care (hours/week)		
1–9 h/week	110	27.0
10–19 h/week	77	18.9
20–34 h/week	57	14.0
35–49 h/week	41	10.1
50 + hours/week	122	30.0
Cared-for persons' characteristics		
Age of CfP, Mean (s.d.)	35.0	(13.9)
Gender of CfP	*n*	%
Male	255	62.7
Female	152	37.3
Primary diagnosis of CfP		
Schizophrenia	170	41.8
Psychosis	202	49.6
Type 1 Bipolar disorder	35	8.6
Time since CfP illness onset		
0–5 years	197	48.4
>5–10 years	64	15.7
>10 years	146	35.9

### Health outcomes of carers

Carers had a mean score of 42.2 (s.d. 9.2) on WEMWBS which ranged between 17 and 68 across the study population. Partners of the CfP had the lowest WEMWBS score (38.9, s.d. 9.5), while carers who were friends or other relatives scored the highest with WEMWBS (47.3, s.d. 9.3). The WEMWBS score was also higher for carers from a BAME ethnicity (45.9, s.d. 9.4) compared to those of White ethnicity (41.7, s.d. 9.1). The mean MAKS score was 23.7 (s.d. 2.9), with the maximum score being 30 this indicated that carers had good mental health knowledge (Evans-Lacko *et al*., 2010). The mean EQ-5D VAS score was 67.6 (s.d. 19.2), where 100 is perfect health, this mean is lower compared to the general population (82.8 s.d. 23.3) for the UK (Szende and Williams, 2004). See Table 2 for a summary of carers' health outcome variables and HSE general population's WEMWBS and EQ-5D index value data.

**Table 2.** Summary of carers' and HSE sample's health outcome variables

Health outcome measures	Score range and indication	Carer sample (*n* = 407)			HSE samples
		Mean (s.d.)	IQR	Median	Mean (s.d.)
WEMWBS	14–70 (best)	42.2 (9.2)	36–49	42	49.9 (10.8)*
MAKS	6–30 (best)	23.7 (2.9)	22–26	24	NA
ECI – Negative subscale	0–208 (worst)	108.5 (32.4)	89–132	110	NA
ECI – Positive subscale	0–56 (best)	30.3 (7.4)	25–35	31	NA
FQ	10–80 (worst)	50.9 (10.4)	44–57	51	NA
CWS – Wellbeing subscale	0 = 128 (best)	73.5 (28.2)	53–97	76	NA
CWS – Support subscale	0–51 (worst)	22.4 (12.0)	14–31	22	NA
EQ-5D-5L visual analogue scale	0–100 (best)	67.6 (19.2)	52–82	70	NA
EQ-5D-5L index value score	0–1 (best)	0.73 (0.23)	0.69–0.88	0.77	0.88 (0.25)**

IQR, interquartile range; WEMWBS, Warwick-Edinburgh Mental Wellbeing Scale; MAKS, Mental Health Knowledge Schedule; ECI, Experience of Caregiving Index; FQ, Family Questionnaire; CWS, Carer Wellbeing and Support Scale; NA, not applicable; *n = 6799 from HSE 2016, **n = 7136 from HSE 2017.

### Comparing carers' mental wellbeing and QoL with HSE samples

**Fig. 1.**
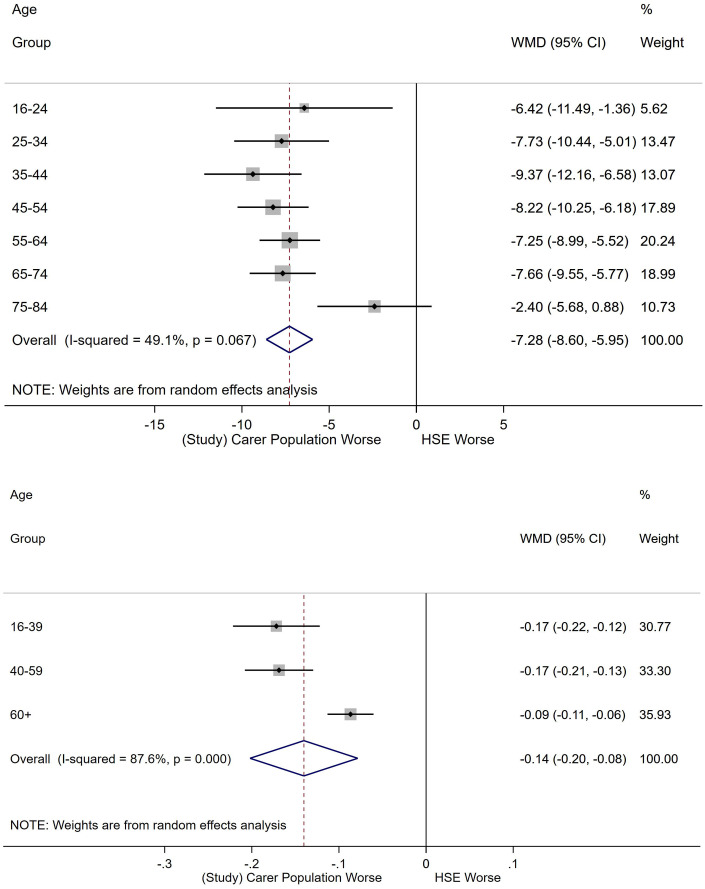
(a) Meta-analysis of WEMWBS score between study carer and HSE samples, (b) meta-analysis of EQ-5D index value score between study carer and HSE samples.

Figure 1*a* depicts the difference in WEMWBS score for carers in this study compared to the general population of the same age range from the most recent HSE data (*n* = 6,799, 51.3% women) (NHS Digital HSE, 2016). The overall weighted pooled WEMWBS score for carers was lower than the HSE sample by −7.3 (95% CI −8.6 to −6.0, *p* < 0.01). These differences are two to three times the MCID of 3 points on WEMWBS (Maheswaran *et al*., 2012). A comparison of EQ-5D index value scores between carers and the HSE general population (*n* = 7136, 51.8% women) (NHS Digital HSE, 2017), estimated a pooled mean difference of −0.14 (−0.2 to −0.1, *p* < 0.01), indicating carers had poorer QoL (see Fig. 1*b*). Index value score differences were largest in the 16–39 age group, −0.17 (−0.2 to −0.1) and smallest for those older than 60, −0.14 (−0.2 to −0.1).

### Correlations between health and caregiving-related outcomes

We proceeded to explore correlations between carers' wellbeing and QoL and other health and caregiving-related outcomes. There were strong negative correlations between mental wellbeing and negative caregiving experience, −0.47 (95% CI −0.5 to −0.4, *p* < 0.01); that is, carers' mental wellbeing was better among those who had a lower ECI negative subscale score. Similarly, carers' mental wellbeing was found to be negatively correlated with EE (−0.55. −0.6 to −0.5, *p* < 0.01); i.e. better wellbeing in those with lower EE scores. Conversely, carers' mental wellbeing was positively correlated with positive caregiving experience, 0.31 (0.2–0.4, *p* < 0.01). Strong positive correlations occurred between mental wellbeing and carer wellbeing, 0.65 (0.6–0.7, *p* < 0.01). Carers' wellbeing, in turn, had strong negative correlations with negative caregiving experience (−0.77, −0.8 to −0.7, *p* < 0.01) and EE (−0.79, −0.8 to −0.7, *p* < 0.01), respectively. Between other health outcomes, strong positive correlations were identified between negative caregiving experience and EE, 0.76 (0.7–0.8, *p* < 0.01).

### Association between carers' characteristics and health outcomes

#### Wellbeing

Mental wellbeing differed between White and BAME carers, with the latter having a higher adjusted WEMWBS score, as reported in Table 3. The relationship with CfP was found to be associated with wellbeing, with partners and friends/other relatives having poorer wellbeing compared to parents. Table 3 depicts that compared with the reference group (carers who were cohabiting), single carers had significantly poorer wellbeing. There was evidence for an interaction between the age of CfP and the relationship with the carer (*p* < 0.01). With the interaction, a year's increase in age of CfP corresponds to an increase in wellbeing for parents (0.3, 0.1–0.5), partners (0.2, 0.0–0.4), and friend/other relatives (0.4, 0.1–0.6), but a decrease (−0.1, −0.3 to 0.1) for children/siblings.

**Table 3.** Selected covariates from multi-variable regression analyses for WEMWBS

Regression coefficient	Partial WEMWBS regression results		
	Estimate	95% confidence interval	*p* value*
Age of CfP	0.29	(0.1–0.5)	*p* < 0.01
Ethnicity			
White	Ref.	–	*p* < 0.01
BAME	4.50	(1.8–7.2)	
Relationship with CfP			
Parent	Ref.	–	*p* = 0.03
Spouse/Partner	−6.83	(−16.9 to 3.3)	
Child/Sibling	10.32	(−1.2 to 21.9)	
Friend/Other relatives	−3.23	(−17.2 to 10.7)	
Marital status			
Married	Ref.	–	*p* < 0.01
Single/Other	−3.56	(−5.6 to −1.5)	
Duration of Care (hours/week)			
1–9	Ref.	–	*p* = 0.07
10–19	−1.25	(−3.8 to 1.3)	
20–34	−2.27	(−5.2 to 0.7)	
35–49	−3.13	(−6.5 to 0.3)	
50 +	−4.29	(−7.2 to −1.3)	
Interaction			
Age of CfP × Spouse/Partner	−0.06	(−0.3 to 0.2)	*p* < 0.01
Age of CfP × Child/Sibling	−0.36	(−0.6 to −0.1)	
Age of CfP × Friend/Other	0.08	(−0.2 to 0.4)	

*p values shown for categorical coefficients are from a Wald test for statistical significance for all levels of coefficient.

These estimates are taken from the full regression model which can be seen in online Supplementary Table 1 with the full models from all outcomes.

Online Supplementary Table 1 shows the multi-variable regression analyses for all health outcomes with 12 covariates in the model. We focused on reporting the associations and interactions with significant results herewith. Focusing on carer-specific wellbeing (i.e. CWS wellbeing scores), we found that time spent caring (*p* = 0.01) has strong negative associations with carer wellbeing. As time spent caring increased, carer's wellbeing deteriorated; compared to those providing 9 or less hpw on caring, those who spent 10–19 hpw and ⩾50 hpw caring had a decrease of −6.0 (−14.0 to 2.0, *p* = 0.14) and −16.6 (−25.8 to −7.5, *p* < 0.01) in CWS wellbeing scores, respectively.

#### Negative caregiving experience and EE

The adjusted ECI negative subscale for the carers reduced by −0.5 (−1.0 to −0.1, *p* = 0.02) for each year increase in age of the CfP. Carers' FQ score also reduced with increasing age of CfP, with a mean decrease of −0.1 (−0.2 to 0.0, *p* = 0.19) for each year, as reported in online Supplementary Table 1. These translate into a reduction in the carers' negative caregiving experience and EE when the CfP was older in age. Compared to parents who spent the same time caring, carers who were partners (3.6, −5.3 to 12.5), siblings/children (3.4, −3.7 to 10.5) had poorer EE (group *p* = 0.03). Those who were single had poorer experience compared to those who were in an intimate relationship, with higher estimated mean ECI-negative scores of 7.3 (0.0–14.6, *p* = 0.05). Spending more than 35 hpw caregiving increased carers' negative caregiving experience significantly; with those caring for 35–49 hpw having the highest ECI negative score increase of 37.8 (8.5–67.1, *p* < 0.01), compared to those caring for <10 hpw, of the same gender and same relationship.

#### Other health and caregiving-related outcomes

No demographic or caregiving characteristics, other than the carer's marital status, showed any strong association with mental health knowledge. Those who were single had an estimated mean reduction in MAKs score of −0.8 (95% CI −1.5 to −0.1, *p* = 0.03). The model for the ECI positive subscale showed that female carers had a score that was 6.7 (3.1–10.3, *p* < 0.01) higher than male carers spending the same time caring. The score was also higher for BAME carers, with an estimated mean increase of 6.0 (3.7–8.3, *p* < 0.01) compared to carers of White ethnicity. Relative to parents who spent the same time caring, children/siblings and friends/other relatives had an increase of 12.0 (95% CI 4.0–20.2, *p* < 0.01) and 12.3 (95% CI 1.7–22.8, *p* = 0.02) in CWS Support score respectively, i.e. less satisfied with the support received. Strong statistical associations were identified between EQ-5D VAS and the following demographics showing: a higher score for those married (*p* < 0.01), higher for retired (*p* < 0.01) and lower for those unemployed compared to those working or in education (*p* < 0.01) (see online Supplementary Table 1).

## Discussion

Our study results identified that psychosis carers have significantly poorer mental wellbeing and QoL than their peers in the wider general population in England of a similar age range. Carers of people with psychosis seemed to have much poorer mental wellbeing than those general carers in the HSE sample who spent more than 10 hpw caring (Bridges, 2012). Partners of the CfP and single carers were found to have the poorest mental wellbeing. Partners were also found to have the lowest carer's wellbeing, worst negative caregiving experience, higher EE and lower satisfaction with support received. Spending over 35 hpw on caring was associated with a significantly poorer negative experience. Increased caregiving over this threshold was also associated with higher EE, indicating that carers were more likely to exhibit critical attitude towards their CfP. All carers, apart from siblings and children, had higher wellbeing, when the individual they cared for was older.

Our study confirms that associations found between carers' characteristics in general and outcomes (Singleton *et al*., 2002; Cooper *et al*., 2010; Bridges, 2012; Smith *et al*., 2014) also apply in the context of psychosis caregiving. Firstly, other researchers suggested that while siblings and children ‘inherit’ the key-carer role from the original carers (most likely their parents who can no longer provide care) (Bowman *et al*., 2014; Sin *et al*., 2016), they tend to step into the dual caring role for the older generation as well as for dependent children. These ‘sandwich carers’ are not widely-recognised in psychosis literature or clinical practice (Ben-Galim and Silim, 2013; Centre for Policy on Ageing, 2015). Secondly, carers who are not in a relationship are likely to be those described as a lone carer having no relief to share the caregiving load while also bearing more socio-economic challenges (Singleton *et al*., 2002; The Schizophrenia Commission, 2012; Sin *et al*., 2017; Carers Trust, 2019). Thirdly, our results showed that partners had worse health outcomes than other carers of individuals with psychosis. The current study might well be the first which has a high proportion of partners as the key carers, contrary to general beliefs that people with psychosis are less likely to have an intimate partner (Office for National Statistics, 2001). The results may also suggest that partners are often the ones who shoulder most of the emotional burden as well as practical caring responsibilities.

In our analysis, BAME carers were found to have better mental wellbeing and a more positive experience of caregiving than those with a White background. However, these results should be interpreted with caution due to the relatively small proportion of BAME in our overall sample. Another salient finding concerns the poorer satisfaction with support received in carers who did not live with the CfP. While these carers provide immense support to their CfP from a distance, they might be less well-recognised by services and professionals as a carer.

### Strengths and limitations

To our knowledge, this study is the first examining wellbeing and QoL of carers supporting individuals with psychosis. Our study sample comes from an England-wide digital intervention trial which prospectively collected high-quality baseline data. A principled modelling approach was used, i.e. models were not fitted to the data but rather pre-specified covariates and interactions were examined. This study was one of the first online trials of a digital intervention targeting carers and its participants may differ systematically from the wider population of carers for people with psychosis. Because of the gender imbalance, the representativeness of our sample cannot be definitely established and caution should be given to the comparison of carers' wellbeing and QoL with HSE general population data. Most carers were recruited through 30 mental health trusts across England indicating their CfP was receiving care at the time of enrolling into the trial. These carers might have increased needs for support, hence were actively seeking help. Only carers gave consent to join the study; the data we collected on their CfP were limited. Our sample only included a small proportion of Black carers, contrary to the well-documented over-representation of people of Black African and Caribbean backgrounds diagnosed with psychosis and having poorer outcomes, yet the least likely to access carer-focused or family-based intervention (The Schizophrenia Commission, 2012; NICE, 2014). This study used a cross-sectional design and thus results imply no causal effects.

### Future research and implications for clinicians and policy makers

All carers of people with psychosis should be under the care of a general practitioner (GP) and in some cases may also be in contact with mental health services and clinicians due to their caring role for their loved ones. We advocate for an increased awareness of carers' needs across healthcare settings. We suggest that all GP practices add the details of carers for individuals with psychosis to the existing carers or SMI register (DOH, 2008, 2014) and screen for their wellbeing to aid early identification of support needs. Further large-scale prospective studies are needed to investigate and confirm associations between carers' health and various demographic and caregiving-related outcomes including negative caregiving experience and EE. These will inform the development of a predictive model to determine risk factors and potential interventions for healthcare professionals to use as a tool. Meanwhile, our study results indicate that there are potential risk factors among carers that clinicians may benefit from paying particular attention to, including partners, lone carers and those providing more than 35 hpw caregiving. These factors identify carers most at risk of poor wellbeing and other health outcomes and also signal their diminishing coping capacity and an increased risk in them being critical to the CfP (Szmukler *et al*., 1996; Cooper *et al*., 2010; Sin *et al*., 2017). Our learning from this study may also be useful to other researchers in devising effective interventions which are easily accessible for carers. Using corroborating evidence from future prospective studies, design and development of future interventions should target the risk factors (e.g. lone carers) and needs (e.g. communication with the CfP) identified in the carers.

## Data Availability

The datasets generated during and/or analysed during the current study are not publicly available due to conditions on participant consent and other ethical restrictions.

## Supplementary material

For supplementary material accompanying this paper visit http://dx.doi.org/10.1017/S2045796020001067. click here to view supplementary material
