# Supplementary material for: Mental health and caregiving experiences of family carers supporting people with psychosis
Source: Epidemiol Psychiatr Sci. 2021 Jan 8;30:e3. doi: 10.1017/S2045796020001067 (PMC7116786; doi:10.1017/S2045796020001067)
Supplement: Supplementary file 1 [file S2045796020001067sup001.docx]

**Supplemental Table 1: Multi-variable regression analyses for all health outcomes with 12 covariates in the model**

| **Regression Coefficient** | **WEMWBS** | **MAKS** | **ECI Positive Subtotal** | **ECI Negative Subtotal** | **CWS - Wellbeing** | **CWS - Support** | **FQ** | **ED-5D-5L VAS** |
| --- | --- | --- | --- | --- | --- | --- | --- | --- |
| **Age of Carer** | -0.18  (-0.8 to 0.5) p=0.58 | -0.07  (-0.3 to 0.1) p=0.48 | -0.06  (-0.5 to 0.4) p=0.81 | 1.00  (-1.0 to 3.0) p=0.32 | -0.27  (-1.9 to 1.4) p=0.75 | -0.04  (-0.8 to 0.7) p=0.91 | 0.38  (-0.3 to 1.0) p=0.25 | -0.73  (-2.1 to 0.6) p=0.29 |
| **Age of Carer Squared** | 0.00  (0.0 to 0.0) p=0.82 | 0.00  0.0 to 0.0) p=0.62 | 0.00  (0.0 to 0.0) p=0.99 | -0.01  (0.0 to 0.0) p=0.42 | 0.00  (0.0 to 0.0) p=0.84 | 0.00  (0.0 to 0.0) p=0.73 | 0.00  (0.1 to 0.0) p=0.20 | 0.01  (0.0 to 0.0) p=0.34 |
| **Age of CfP** | **0.29**  **(0.1 to 0.5) p=0.002** | 0.00  (0.0 to 0.0) p=0.86 | 0.00  (-0.1 to 0.1) p=0.97 | **-0.54**  **(-1.0 to -0.1) p=0.02** | **0.38**  **(0.0 to 0.8)**  **p=0.04** | -0.14  (-0.3 to 0.0) p=0.10 | -0.10  (-0.2 to 0.1) p=0.19 | 0.14  (-0.3 to 0.5) p=0.48 |
| **Gender** | p=0.30 | p=0.82 | p<0.01 | p=0.49 | p=0.39 | p=0.13 | p=0.76 | p=0.47 |
| Male | Ref | Ref | Ref | Ref | Ref | Ref | Ref | Ref |
| Female | 1.23  (-1.1 to 3.6) | 0.09  (-0.7 to 0.9) | **6.67**  **(3.1 to 10.3)** | 5.44  (-9.9 to 20.8) | -3.23  (-10.5 to 4.1) | 2.47  (-0.7 to 5.7) | 0.45  (-2.4 to 3.3) | -1.82  (-6.8 to 3.1) |
| **Gender of CfP** | p=0.23 | p=0.14 | p=0.21 | p=0.36 | p=0.34 | p=0.99 | p=0.93 | p=0.48 |
| Male | Ref | Ref | Ref | Ref | Ref | Ref | Ref | Ref |
| Female | 1.23  (-1.1 to 3.6) | 0.09  (-0.7 to 0.9) | 6.67  (3.1 to 10.3) | 5.44  (-9.9 to 20.8) | -3.23  (-10.5 to 4.1) | 2.47  (-0.7 to 5.7) | 0.45  (-2.4 to 3.3) | -1.82  (-6.8 to 3.1) |
| **Ethnicity** | p<0.01 | p=0.33 | p<0.01 | p=0.99 | p=0.51 | p=0.08 | p=0.15 | p=0.13 |
| White | Ref | Ref. | Ref. | Ref. | Ref. | Ref. | Ref. | Ref. |
| BAME | **4.50**  **(1.8 to 7.2)** | 0.47  (-0.5 to 1.4) | **5.99**  **(3.7 to 8.3)** | 0.06  (-9.9 to 10.0) | 2.87  (-5.7 to 11.4) | *-3.29*  *(-7.0 to 0.5)* | -2.40  (-5.7 to 0.9) | 4.44  (-1.3 to 10.2) |
| **Relationship with CfP** | p=0.03 | p=0.58 | p=0.32 | p=0.05 | p<0.01 | p<0.01 | p=0.03 | p=0.12 |
| Parent | Ref | Ref | Ref | Ref | Ref | Ref | Ref | Ref |
| Spouse/Partner | **-6.83**  **(-16.9 to 3.3)** | -0.95  (-2.4 to 0.5) | -3.18  (-6.7 to 0.3) | **3.57**  **(-23.4 to 30.5)** | **-14.69**  **(-27.5 to -1.9)** | **3.57**  **(-6.6 to 13.7)** | **3.62**  **(-5.3 to 12.5)** | -10.02  (-35.6 to 15.6) |
| Child/Sibling | **10.32**  **(-1.2 to 21.9)** | -0.58  (-2.5 to 1.3) | -2.36  (-6.9 to 2.2) | **7.35**  **(-14.0 to 28.7)** | **-12.82**  **(-29.8 to 4.2)** | **12.09**  **(4.0 to 20.2)** | **3.40**  **(-3.7 to 10.5)** | 17.08  (-7.8 to 41.9) |
| Friend/Other relative | **-3.23**  **(-17.2 to 10.7)** | -0.65  (-2.5 to 1.2) | -1.07  (-5.5 to 3.3) | **-30.46**  **(-58.3 to -2.6)** | **8.02**  **(-8.2 to 24.3)** | **12.25**  **(1.7 to 22.8)** | **-9.53**  **(-18.8 to -0.3)** | 22.03  (-10.1 to 54.2) |
| **Employment Status** | p=0.13 | p=0.81 | p=0.53 | p=0.33 | p<0.01 | p=0.74 | p=0.51 | p<0.01 |
| Work/Education | Ref | Ref | Ref | Ref | Ref | Ref | Ref | Ref |
| Not Working | -2.10  (-4.4 to 0.2) | -0.19  (-1.0 to 0.6) | 0.64  (-1.3 to 2.6) | 0.83  (-7.5 to 9.1) | **-10.04**  **(-17.2 to -2.9)** | 1.18  (-1.9 to 4.3) | 1.47  (-1.3 to 4.2) | **-12.14**  **(-16.9 to -7.3)** |
| Retired | 0.94  (-2.2 to 4.1) | -0.29  (-1.4 to 0.8) | 1.39  (-1.3 to 4.1) | -8.28  (-19.8 to 3.2) | **4.27**  **(-5.5 to 14.0)** | -0.14  (-4.4 to 4.2) | -0.50  (-4.3 to 3.3) | **4.74**  **(-1.9 to 11.4)** |
| **Highest education achieved** | p=0.19 | p=0.61 | p=0.66 | p=0.35 | p=0.48 | p=0.36 | p=0.16 | p=0.88 |
| Pre-university | Ref | Ref | Ref | Ref | Ref | Ref | Ref | Ref |
| Undergraduate/  Professional | 1.25  (-0.9 to 3.4) | 0.47  (-0.3 to 1.2) | -0.63  (-2.4 to 1.2) | -3.69  (-11.6 to 4.2) | 0.54  (-6.2 to 7.2) | 1.23  (-1.7 to 4.2) | 0.55  (-2.1 to 3.2) | -1.27  (-5.9 to 3.4) |
| Postgraduate | -1.24  (-3.7 to 1.3) | 0.26  (-0.6 to 1.1) | -0.27  (-2.4 to 1.8) | 2.22  (-6.9 to 11.3) | -2.54  (-10.3 to 5.2) | 2.93  (-0.5 to 6.4) | 1.54  (-1.5 to 4.5) | -2.09  (-7.4 to 3.2) |
| Apprenticeship | -0.14  (-3.3 to 3.0) | 0.50  (-0.6 to 1.6) | 0.99  (-1.7 to 3.6) | 4.88  (-6.7 to 16.5) | -6.31  (-16.1 to 3.5) | 2.34  (-2.0 to 6.7) | 4.19  (0.4 to 8.0) | -1.70  (-8.5 to 5.1) |
| **Marital status** | p<0.01 | p=0.03 | p=0.16 | p=0.05 | p<0.01 | p<0.01 | p=0.06 | p=0.03 |
| Married | Ref | Ref | Ref | Ref | Ref | Ref | Ref | Ref |
| Single/Other | **-3.56**  **(-5.6 to -1.5)** | **-0.76**  **(-1.5 to -0.1)** | -1.19  (-2.9 to 0.5) | **7.33**  **(0.0 to 14.6)** | **-11.16**  **(-17.3 to -5.0)** | **3.53**  **(0.8 to 6.3)** | *2.33*  *(-0.1 to 4.7)* | **-6.50**  **(-10.8 to -2.2)** |
| **Living arrangement** | p=0.13 | p=0.59 | p=0.30 | p=0.01 | p=0.37 | p=0.02 | p=0.24 | p=0.61 |
| With CfP | Ref | Ref | Ref | Ref | Ref | Ref | Ref | Ref |
| Not with CfP | -1.84  (-4.3 to 0.6) | -0.23  (-1.1 to 0.6) | -1.09  (-3.1 to 1.0) | **11.42**  **(2.4 to 20.4)** | -3.39  (-10.9 to 4.1) | **3.91**  **(0.6 to 7.3)** | 1.75  (-1.2 to 4.7) | -1.37  (-6.6 to 3.9) |
| **Disease of CfP** | p=0.17 | p=0.99 | p=0.71 | p=0.48 | p=0.42 | p=0.34 | p=0.09 | p=0.03 |
| Schizophrenia | Ref | Ref | Ref | Ref | Ref | Ref | Ref | Ref |
| Psychosis | -0.09  (-2.3 to 2.1) | -0.02  (-0.8 to 0.7) | 0.13  (-1.7 to 2.0) | -2.78  (-10.8 to 5.2) | 3.35  (-3.5 to 10.1) | -1.35  (-4.3 to 1.6) | *-1.19*  *(-3.8 to 1.4)* | **2.53**  **(-2.1 to 7.1)** |
| Type 1 Bipolar | -2.99  (-6.3 to 0.3) | 0.06  (-1.1 to 1.2) | 1.15  (-1.6 to 3.9) | 4.41  (-7.6 to 16.5) | -2.83  (-13.0 to 7.3) | 1.87  (-2.6 to 6.4) | *3.25*  *(-0.7 to 7.2)* | **-6.89**  **(-13.9 to 0.1)** |
| **Duration of Care**  **(hours/week)** | p=0.07 | p=0.49 | p=0.04 | P<0.01 | P<0.01 | p=0.41 | p=0.24 | p=0.43 |
| 1-9 | Ref | Ref | Ref | Ref | Ref | Ref | Ref | Ref |
| 10-19 | *-1.25*  *(-3.8 to 1.3)* | -0.02  (-0.9 to 0.9) | **5.26**  **(0.1 to 10.5)** | **-9.97**  **(-33.7 to 13.8)** | **-5.98**  **(-14.0 to 2.0)** | 2.14  (-2.1 to 6.4) | 2.45  (-1.2 to 6.1) | -4.61  (-11.1 to 1.9) |
| 20-34 | *-2.27*  *(-5.2 to 0.7)* | 0.66  (-0.4 to 1.7) | **6.67**  **(1.1 to 12.2)** | **-3.23**  **(-29.9 to 23.5)** | **-11.00**  **(-20.2 to -1.8)** | 4.24  (-0.7 to 9.1) | 1.61  (-2.7 to 5.9) | -3.99  (-11.5 to 3.6) |
| 35-49 | *-3.13*  *(-6.5 to 0.3)* | -0.21  (-1.4 to 1.0) | **7.75**  **(1.6 to 13.9)** | **37.77**  **(8.5 to 67.1)** | **-15.47**  **(-26.0 to -4.9)** | -0.59  (-6.6 to 5.4) | 3.55  (-1.7 to 8.8) | -8.14  (-17.4 to 1.1) |
| 50+ | *-4.29*  *(-7.2 to -1.3)* | 0.49  (-0.5 to 1.5) | **6.28**  **(1.6 to 10.9)** | **22.99**  **(1.1 to 44.8)** | **-16.60**  **(-25.8 to -7.4)** | 2.86  (-1.8 to 7.5) | 3.67  (-0.4 to 7.7) | -3.79  (-10.9 to 3.4) |
| **Time since onset** | p=0.52 | p=0.37 | p=0.04 | p=0.39 | p=0.81 | p=0.06 | p=0.88 | p=0.16 |
| 0-5 | Ref | Ref | Ref | Ref | Ref | Ref | Ref | Ref |
| 5-10 | 0.05  (-2.5 to 2.6) | -0.40  (-1.3 to 0.5) | **-2.21**  **(-4.4 to -0.1)** | 4.43  (-4.8 to 13.7) | -1.96  (-9.9 to 6.0) | *3.57*  *(0.1 to 7.0)* | 0.32  (-2.7 to 3.4) | 0.17  (-5.2 to 5.5) |
| 10+ | -1.32  (-3.8 to 1.2) | -0.59  (-1.5 to 0.3) | **0.80**  **(-1.3 to 2.9)** | 5.94  (-3.2 to 15.1) | -2.25  (-10.0 to 5.5) | *3.33*  *(-0.1 to 6.8)* | 0.79  (-2.2 to 3.8) | -4.76  (-10.1 to 0.6) |
| Constant | 46.07  (27.8 to 64.3) | 26.25  (21.6 to 30.9) | 27.60  (16.0 to 39.2) | 76.58  (25.6 to 127.5) | 93.09  (50.8 to 135.4) | 13.73  (-5.2 to 32.6) | 41.53  (24.9 to 58.1) | 95.04  (55.9 to 134.2) |
| **Interactions** | p<0.01 |  |  |  |  |  |  | p=0.11 |
| Age of CfP x Spouse/Partner | **-0.06**  **(-0.3 to 0.2)** |  |  |  |  |  |  | 0.08  (-0.4 to 0.6) |
| Age of CfP x Child/Sibling | **-0.36**  **(-0.6 to -0.1)** |  |  |  |  |  |  | -0.46  (-1.0 to 0.1) |
| Age of CfP x Friend/Other | **0.08**  **(-0.2 to 0.4)** |  |  |  |  |  |  | -0.35  (-1.0 to 0.3) |
|  |  |  | p=0.15 | p=0.09 |  |  |  |  |
| Female x  10-19 hpw |  |  | -4.91  (-10.6 to 0.8) | *19.29*  *(-5.5 to 44.1)* |  |  |  |  |
| Female x  20-34 hpw |  |  | -5.84  (-11.9 to 0.2) | *12.36*  *(-14.7 to 39.5)* |  |  |  |  |
| Female x  35-49 hpw |  |  | -5.37  (-12.1 to 1.3) | *-18.76*  *(-47.7 to 10.2)* |  |  |  |  |
| Female x  50+ hpw |  |  | -5.76  (-10.6 to -0.9) | *-7.30*  *(-28.5 to 13.9)* |  |  |  |  |
|  |  |  |  | p=0.07 |  | p=0.06 | p=0.05 | p=0.15 |
| Spouse/Partner  x 10-19 hpw |  |  |  | *-5.96*  *(-37.4 to 25.4)* |  | *7.74*  *(-4.0 to 19.5)* | *-3.23*  *(-13.5 to 7.0)* | 0.44  (-17.6 to 18.5) |
| Child/Sibling  x 10-19 hpw |  |  |  | *1.13*  *(-25.4 to 27.7)* |  | *-12.43*  *(-22.5 to -2.4)* | *-6.97*  *(-15.8 to 1.8)* | 24.63  (9.1 to 40.1) |
| Friend/Other  x 10-19 hpw |  |  |  | *59.26*  *(19.8 to 98.8)* |  | *4.87*  *(-10.0 to 19.7)* | *17.28*  *(4.2 to 30.3)* | 1.49  (-21.5 to 24.5) |
| Spouse/Partner  x 20-34 hpw |  |  |  | *19.59*  *(-11.7 to 50.9)* |  | *3.39*  *(-8.4 to 15.2)* | *0.29*  *(-10.0 to 10.6)* | -5.26  (-23.5 to 13.0) |
| Child/Sibling  x 20-34 hpw |  |  |  | *18.38*  *(-9.8 to 46.6)* |  | *-7.79*  *(-18.4 to 2.9)* | *-0.92*  *(-10.3 to 8.4)* | 4.43  (-12.1 to 21.0) |
| Friend/Other  x 20-34 hpw |  |  |  | *2.82*  *(-51.5 to 57.2)* |  | *-16.42*  *(-35.7 to 2.9)* | *-8.92*  *(-25.9 to 8.0)* | 20.97  (-8.9 to 50.8) |
| Spouse/Partner  x 35-49 hpw |  |  |  | *-10.02*  *(-40.8 to 20.8)* |  | *9.07*  *(-2.5 to 20.7)* | *-3.56*  *(-13.7 to 6.6)* | -3.24  (-21.2 to 14.7) |
| Child/Sibling  x 35-49 hpw |  |  |  | *0.96*  *(-46.5 to 48.4)* |  | *-15.44*  *(-33.3 to 2.4)* | *10.42*  *(-5.3 to 26.1)* | 12.13  (-15.5 to 39.8) |
| Friend/Other  x 35-49 hpw |  |  |  | *20.91*  *(-24.8 to 66.6)* |  | *-8.58*  *(-25.8 to 8.6)* | *6.30*  *(-8.8 to 21.4)* | 8.54  (-18.0 to 35.1) |
| Spouse/Partner  x 50+ hpw |  |  |  | *11.13*  *(-15.0 to 37.3)* |  | *2.21*  *(-7.6 to 12.0)* | *0.26*  *(-8.3 to 8.9)* | -3.65  (-18.8 to 11.5) |
| Child/Sibling  x 50+ hpw |  |  |  | *0.00*  *(empty)* |  | *0.00*  *(empty)* | *0.00*  *(empty)* | 0.00  (empty) |
| Friend/Other  x 50+ hpw |  |  |  | *-1.94*  *(-52.2 to 48.4)* |  | *-8.13*  *(-27.0 to 10.7)* | *-4.37*  *(-20.9 to 12.2)* | -18.67  (-47.7 to 10.4) |

WEMWBS: Warwick-Edinburgh Mental Wellbeing Scale, MAKS: Mental Health Knowledge Schedule, ECI: Experience of Caregiving Inventory, CWS: Carer Wellbeing and Support Scale, FQ: Family Questionnaire, EQ-5D-5L VAS: EQ-5D-5L Visual Analogue Scale, Ref: Reference, values in blankets: 95% confident internal, p: probability, CfP: Cared -for person, BAME: Black, Asian, and minority ethnic, hpw: hours per week, Variables highlighted in bold-print are those with a p<0.05, Variables highlighted in italics print are those where 0.05≤p<0.10
